# Retrospective quantification of clinical abdominal DCE-MRI using pharmacokinetics-informed deep learning: a proof-of-concept study

**DOI:** 10.3389/fradi.2023.1168901

**Published:** 2023-09-04

**Authors:** Chaowei Wu, Nan Wang, Srinivas Gaddam, Lixia Wang, Hui Han, Kyunghyun Sung, Anthony G. Christodoulou, Yibin Xie, Stephen Pandol, Debiao Li

**Affiliations:** ^1^ Biomedical Imaging Research Institute, Cedars-Sinai Medical Center, Los Angeles, CA, United States; ^2^ Department of Bioengineering, University of California, Los Angeles, Los Angeles, CA, United States; ^3^ Radiology Department, Stanford University, Stanford, CA, United States; ^4^ Division of Digestive and Liver Diseases, Cedars-Sinai Medical Center, Los Angeles, CA, United States; ^5^ Department of Radiological Sciences, University of California, Los Angeles, Los Angeles, CA, United States

**Keywords:** dynamic contrast-enhanced MRI, abdomen, deep learning, quantitative imaging, pharmacokinetic model

## Abstract

**Introduction:**

Dynamic contrast-enhanced (DCE) MRI has important clinical value for early detection, accurate staging, and therapeutic monitoring of cancers. However, conventional multi-phasic abdominal DCE-MRI has limited temporal resolution and provides qualitative or semi-quantitative assessments of tissue vascularity. In this study, the feasibility of retrospectively quantifying multi-phasic abdominal DCE-MRI by using pharmacokinetics-informed deep learning to improve temporal resolution was investigated.

**Method:**

Forty-five subjects consisting of healthy controls, pancreatic ductal adenocarcinoma (PDAC), and chronic pancreatitis (CP) were imaged with a 2-s temporal-resolution quantitative DCE sequence, from which 30-s temporal-resolution multi-phasic DCE-MRI was synthesized based on clinical protocol. A pharmacokinetics-informed neural network was trained to improve the temporal resolution of the multi-phasic DCE before the quantification of pharmacokinetic parameters. Through ten-fold cross-validation, the agreement between pharmacokinetic parameters estimated from synthesized multi-phasic DCE after deep learning inference was assessed against reference parameters from the corresponding quantitative DCE-MRI images. The ability of the deep learning estimated parameters to differentiate abnormal from normal tissues was assessed as well.

**Results:**

The pharmacokinetic parameters estimated after deep learning have a high level of agreement with the reference values. In the cross-validation, all three pharmacokinetic parameters (transfer constant $K^{\mathrm{trans}}$, fractional extravascular extracellular volume $v_{e}$, and rate constant $k_{\mathrm{ep}}$) achieved intraclass correlation coefficient and *R*^2^ between 0.84–0.94, and low coefficients of variation (10.1%, 12.3%, and 5.6%, respectively) relative to the reference values. Significant differences were found between healthy pancreas, PDAC tumor and non-tumor, and CP pancreas.

**Discussion:**

Retrospective quantification (RoQ) of clinical multi-phasic DCE-MRI is possible by deep learning. This technique has the potential to derive quantitative pharmacokinetic parameters from clinical multi-phasic DCE data for a more objective and precise assessment of cancer.

## 1. Introduction

Pharmacokinetic parameters estimated from quantitative DCE-MRI provide quantitative measurements of tissue perfusion and flow characteristics and are promising imaging biomarkers to help differentiate tumor stages and assess prognosis before treatment (1, 2). However, quantitative analysis of clinical multi-phasic abdominal DCE-MRI has been limited (3) due to low temporal resolution (approximately 30 s) with a small number of phases (e.g., *n* = 4).These limitations result from the intrinsic trade-off of MRI between spatial resolution, temporal resolution, and spatial coverage, as well as the need for breath-holding in abdominal MRI. Such low temporal resolution constraints hinder the accurate tracking of dynamic contrast changes, necessitating improved methods for temporal interpolation to enable precise pharmacokinetic parameter estimation.

Semi-quantitative or quantitative analyses with a population-based arterial input function (AIF) are possible (4, 5). However, these methods are subject to various factors that affect signal intensities such as the specific scanner and imaging protocol. While qualitative analysis typically categorizes the type of enhancement curve, quantitative analysis uses mathematical models to derive precise parameters describing physiological processes such as blood flow, permeability, and extravascular extracellular space volume. Although advanced techniques such as multiparametric MRI (mpMRI) (6) and Multitasking DCE (7) have sufficient temporal resolution for quantification, they require special sequences and reconstruction pipelines that are not routinely available on clinical systems. Therefore, parametric quantification based on conventional DCE-MRI is highly desirable, as it has the potential to improve DCE-MRI applications without changing the clinical imaging protocols.

In the last few years, deep learning (DL) techniques have been actively explored for applications in medical imaging (8), such as image registration, spatial super-resolution, denoising, and disease prediction. Several studies investigated the potential of applying DL to DCE-MRI, including tumor segmentation (9), therapy response prediction for cancer (10), lesion malignancy classification (11), and pharmacokinetic parameter estimation from time-resolved DCE-MRI (12–17). These studies demonstrated the capability of DL to extract information and simplify post-processing, as well as the growing interest in pharmacokinetic modeling. However, most of these studies were conducted in qualitative or semi-quantitative DCE-MRI, which will suffer from the limitations of the qualitative or semi-quantitative analysis. Studies conducted in quantitative time-resolved DCE-MRI might not be directly translated into clinical application since most standard-of-care protocols still adopt multi-phasic DCE-MRI.

In this work, we describe a novel quantification method for multi-phasic abdominal DCE-MRI from standard-of-care protocols, retrospectively improving temporal resolution via pharmacokinetics-informed deep learning. Multi-phasic abdominal DCE-MRI was synthesized from Multitasking DCE acquisitions, a novel quantitative DCE technique capable of 2-s temporal resolution (7). The proposed method was able to recover 2-s temporal resolution DCE-MRI and estimate pharmacokinetic parameters from the synthesized multi-phasic data. The agreement of the estimated pharmacokinetic parameters (transfer constant $K^{\mathrm{trans}}$, fractional extravascular extracellular volume $v_{e}$, and rate constant $k_{\mathrm{ep}}$) was assessed against the references estimated from 2-s temporal resolution Multitasking DCE. The ability of pharmacokinetic parameters estimated using synthesized multi-phasic abdominal DCE-MRI to differentiate abnormal from normal tissues was examined. By addressing the low temporal resolution challenge, this work paves the way for broader clinical applicability of quantitative analysis, enhancing the utility of standard DCE-MRI protocols in tumor assessment.

## 2. Materials and methods

The work hypothesized that pharmacokinetics-informed deep learning could recover high-temporal-resolution DCE-MRI from clinical multi-phasic DCE-MRI, which may allow accurate estimation of pharmacokinetic parameters. Forty-five subjects underwent Multitasking DCE-MRI. Multi-phasic DCE-MRI data were synthesized by downsampling the 2-s temporal-resolution Multitasking DCE data to match the clinical imaging protocol. A deep learning pharmacokinetic quantification network was pre-trained by simulation, then a pharmacokinetics-informed deep learning network was trained to improve the temporal resolution for pharmacokinetic parameter estimation. We name the whole proposed methodology as retrospective quantification (RoQ). The performance of kinetic parameters estimated using synthetic multi-phasic DCE-MRI based on deep learning was assessed in two aspects: (1) agreement against the 2-s temporal-resolution reference. (2) capability in differentiating pancreas in healthy subjects and patients with pathologically confirmed pancreatic ductal adenocarcinoma (PDAC) and chronic pancreatitis (CP). Implementation code of the proposed method was made available asopen-source at https://github.com/LockyChao/RoQ-DCE-DL/tree/master.

### 2.1. In-vivo data acquisition and synthesized data generation

#### 2.1.1. In vivo data acquisition

The *in vivo* study was approved by the local institutional review board. Written informed consent was obtained in all subjects. Forty-five subjects were imaged, including 22 healthy volunteers, 14 patients with pathologically confirmed PDAC, and 9 patients with chronic pancreatitis.

All imaging data used in training and validation were collected on a 3 T clinical MR scanner (Biograph mMR, Siemens Medical Solutions, Erlangen, Germany). The routine protocol for pancreas and tumor delineation was acquired first. It included 3D T1W volumetric interpolated breath-hold examination (VIBE) with Dixon fat suppression, multi-slice T2W half-Fourier acquisition single-shot turbo spin echo (HASTE), a multi-slice single-shot (SS) EPI DWI, and a 2D MOLLI T1-mapping sequence. A six-dimensional free-breathing Multitasking sequence was used for DCE MRI (7). 0.1 mmol/kg gadolinium-based contrast media (Gadavist, 0.1 mmol/kg; Bayer Schering Pharma, Berlin, Germany) was administered 3 min after the start of data acquisition with an injection rate of 2 ml/s. Multitasking imaging parameters include FOV = 380 × 268 mm^2^, spatial resolution = 1.4 mm × 1.4 mm × 6.0 mm, number of slices = 48, TR/TE = 5.60/2.45 ms, flip angle = 10°, and a total imaging time of 12 min. Image reconstruction and post-processing were performed offline by the built pipeline in our prior work (7) in MATLAB (R2018a, MathWorks, Natick, MA), resulting in dynamic T1 maps at 2-s temporal resolution. The pancreas and tumors were manually delineated by an experienced radiologist.

#### 2.1.2. Clinical abdominal multi-phasic DCE data synthesis

**Figure 1:**
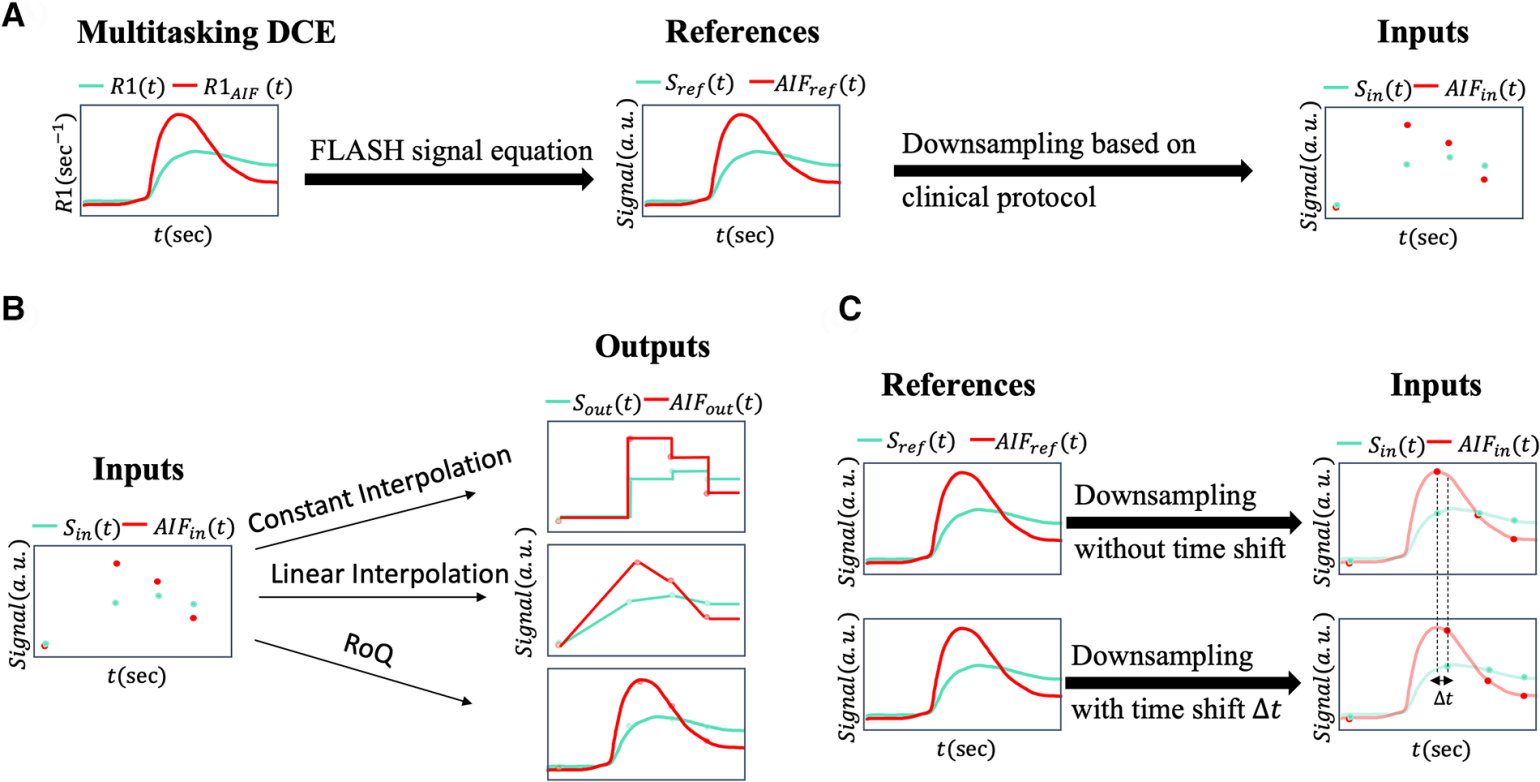
(**A**) This figure illustrates the data preprocessing pipeline. The T1W DCE-MRI signal $S_{\mathrm{ref}}$ and arterial input function $\mathrm{AI}F_{\mathrm{ref}}$ are generated from 2-s temporal-resolution R1 from Multitasking DCE data via the FLASH signal equation. These signals were downsampled to multi-phasic DCE-MRI signals $S_{\mathrm{in}}$ and $\mathrm{AI}F_{\mathrm{in}}$ according to the clinical protocol. During the neural network training process, $S_{\mathrm{in}}$ or $\mathrm{AI}F_{\mathrm{in}}$ served as inputs and $S_{\mathrm{ref}}$ or $\mathrm{AI}F_{\mathrm{ref}}$ served as reference signals. (**B**) Different temporal interpolation methods. The synthesized multi-phasic DCE-MRI $S_{\mathrm{in}}$ (left) was temporally interpolated by constant interpolation (right upper), where the time steps between two clinical phases were set to be the phase before time step; by linear interpolation (right middle), where the time steps between two clinical phases were linearly interpolated; by RoQ (right bottom), where the curves were obtained from the proposed temporal super-resolution method. (**C**) Simulation pipeline for the bolus arrival time displacement. Upper figure showed the downsampling without time shift, where the first post-contrast phase was sampled at the AIF peak. Lower figure showed the scenario with time shift, where first post-contrast phase was sampled with displacement Δt to the AIF peak. The temporal resolution and number of phases were kept the same.

**Figure 2:**
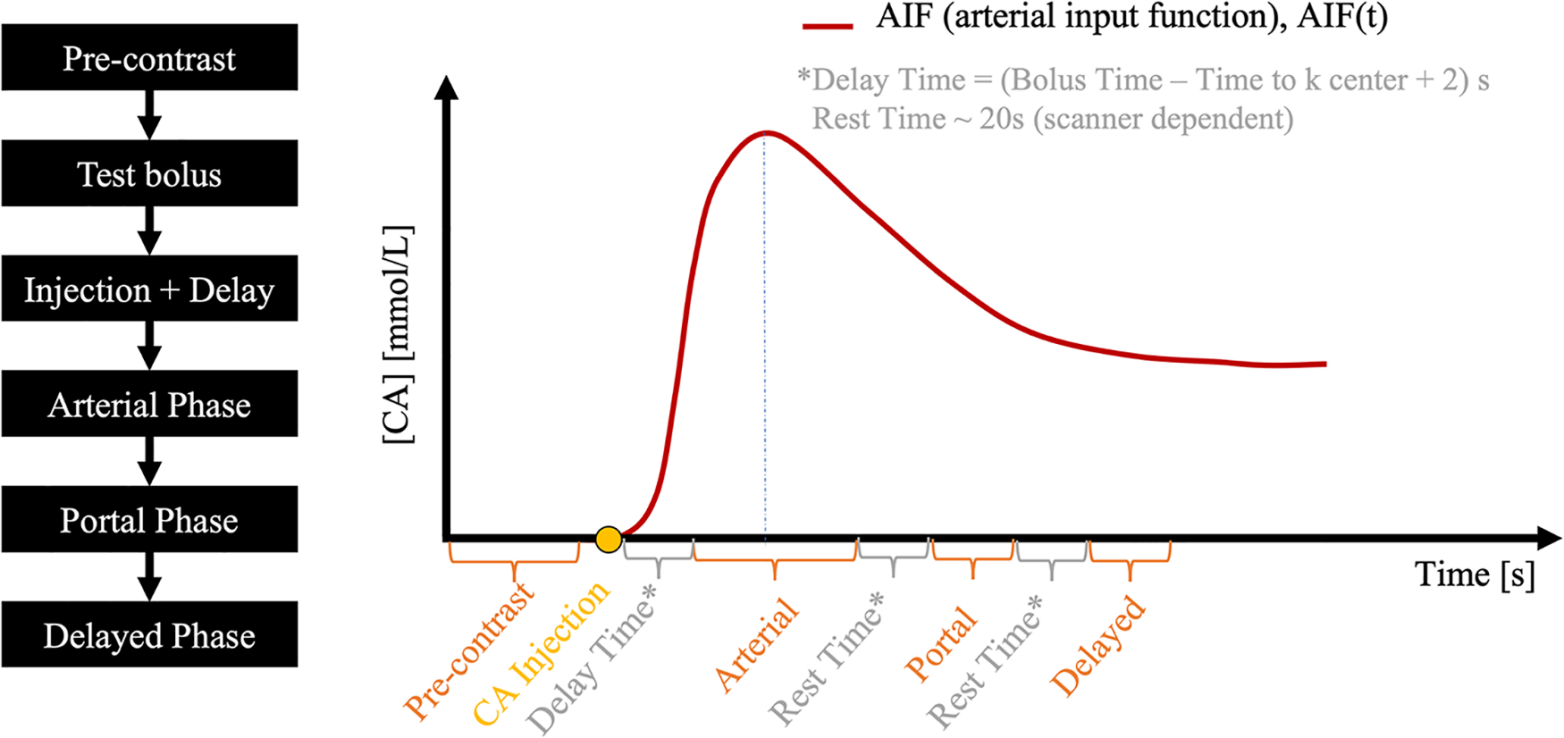
A typical abdominal DCE-MRI protocol. The pre-contrast phase is followed by a test bolus, from which the contrast media bolus delay time is measured and then used to time the subsequent arterial phase. Portal and delayed phases are acquired after respiratory rest time gaps.

The data preprocessing pipeline is illustrated in Figure 1A. Multitasking DCE generates dynamic T1 maps whereas clinical DCE-MRI acquires T1W images. The FLASH signal equation was therefore used to convert high-temporal resolution T1 maps to high-temporal resolution T1W signals, which served as the reference for deep learning. Multi-phasic DCE-MRI was synthesized from a high-temporal resolution T1W signal based on a typical clinical abdominal DCE-MRI protocol (18), as shown in Figure 2.

Abdominal multi-phasic DCE-MRI has one pre-contrast and three post-contrast phases: arterial, portal, and delayed enhancement. A test bolus scan is usually acquired after the pre-contrast phase to estimate contrast agent arrival time to the region of interest, which is used to time the start of the arterial phase in a full-dose DCE scan, such that the central k-space is acquired at peak arterial contrast agent concentration. Subsequent post-contrast phases are timed based on the clinical protocol used at our institution, resulting in a 30-s temporal resolution (10-s acquisition and 20-s gap).

The standard Tofts model was used for pharmacokinetic modeling in the pancreas, as it is well suited to PDAC (19). To linearly convert T1W signal intensity to contrast agent concentration for parametric quantification, it was baseline-corrected and normalized by the pre-contrast signal $S_{0}$.

**Figure 3:**
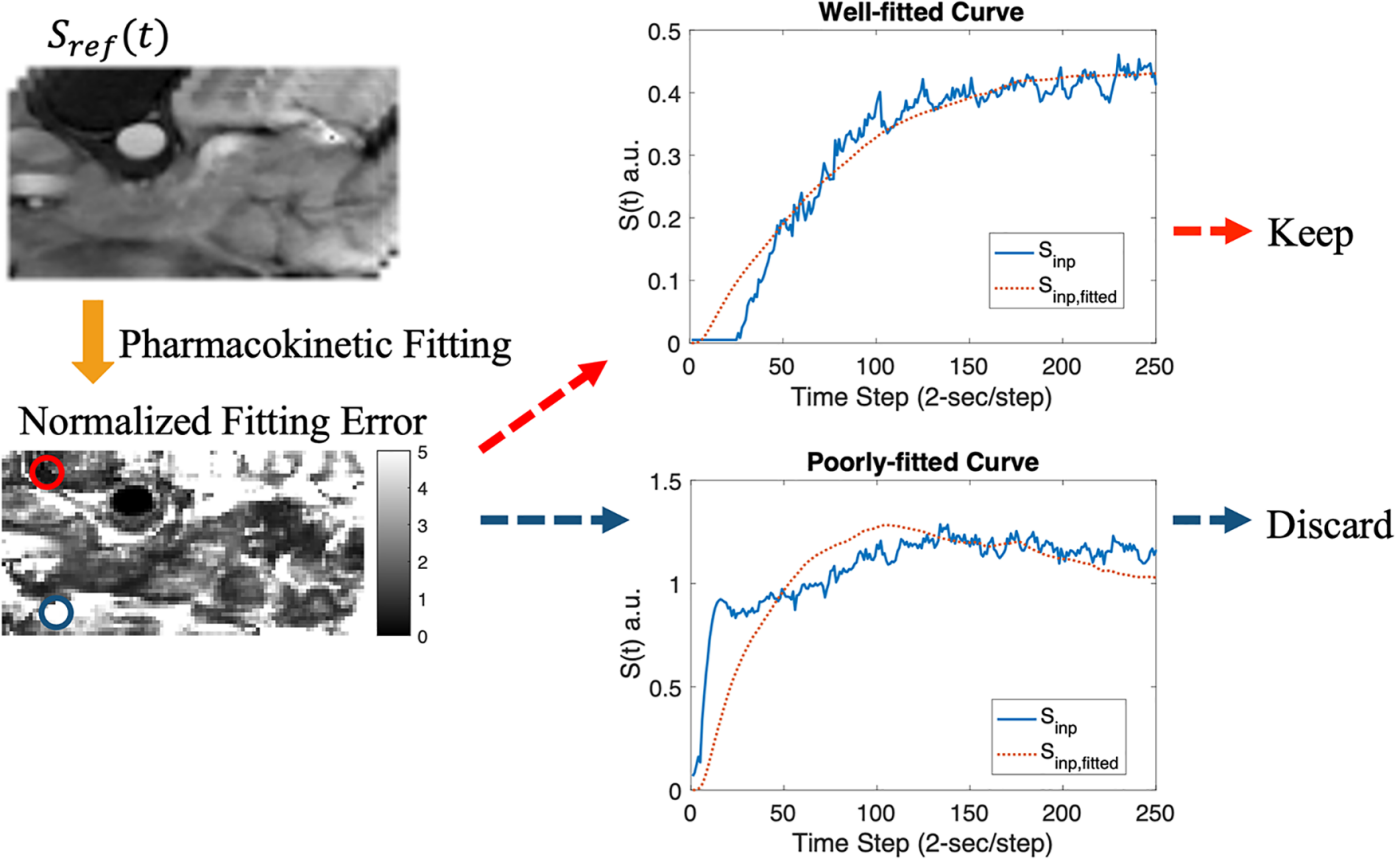
Data screening pipeline. The synthesized T1W DCE-MRI $S_{\mathrm{ref}}$ (upper left) was fitted using the standard Tofts model, resulting in a normalized fitting error map (lower left). Example plots for well-fitted (red circle) and poorly-fitted pixels (blue circle) are shown.

DL-based DCE quantification was performed on a voxel-by-voxel basis. Because that the standard Tofts model is not appropriate for all tissues, especially voxels with high noise level, voxels were pre-screened based on the normalized fitting error of the standard Tofts model, i.e., by dividing the fitting error by peak signal intensity (Figure 3). This also removed voxels with low SNR which may not be useful inputs for training. An empirical threshold of 0.7 was chosen as a trade-off of dataset size and data quality, resulting in 57,891 voxels for training the DL model. A total time step of 130 or a total time duration of 260-s in the DCE time course was used.

#### 2.1.3. Low-temporal resolution data synthesis

We utilized two temporal interpolation methods from low temporal resolution data for comparison. Illustrated in Figure 1B, the first interpolation method, referred to as constant interpolation, applied a step-like approach, creating a distinct separation between each phase. The second method implemented linear interpolation, where each pair of neighboring phases formed a linear segment, providing a smoother transition between the stages. These methods allowed for comparisons of different strategies for handling low-temporal resolution data, thus assessing their impact on the analysis.

### 2.2. The pharmacokinetics quantification network

**Figure 4:**
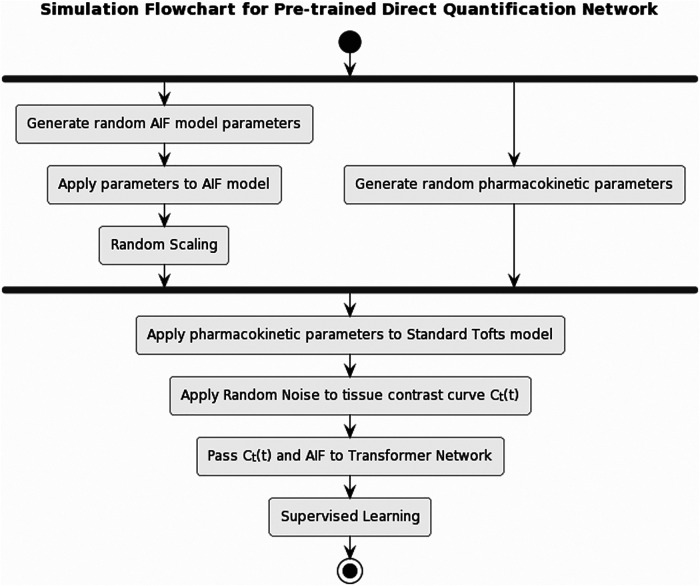
Pipeline for pre-training the pharmacokinetics quantification network. AIF was simulated by applying randomly generated population-based AIF model parameters, then randomly scaled. A set of pharmacokinetic parameters was randomly generated, which was applied to standard Tofts model to obtain tissue contrast curve $C_{t}(t)$. Gaussian noise was then applied to $C_{t}(t)$. Afterwards, $C_{t}(t)$ and AIF were passed to the network to learn the pharmacokinetic parameters.

Inspired by existing research (14, 15) on the direct estimation of pharmacokinetics, we developed a pharmacokinetics quantification network. Trained by simulation prior to the temporal super-resolution network, this network provides pharmacokinetics constraints during training and enables a more efficient quantification process. Figure 4 illustrates the simulation flowchart. Random arterial input function (AIF) model parameters (20) were generated to create a population-based AIF curve, which was then randomly scaled for data augmentation. We generated random pharmacokinetic parameters within a physically reasonable range ($K^{\mathrm{trans}}$: 0.01–12 1/min, $k_{\mathrm{ep}}$:0.01–24 1/min, $v_{e}$: 0.1–0.8) using the standard Tofts model to obtain the tissue contrast curve $\;C_{t}(t)$. Gaussian noise was added to $C_{t}(t)$, then $C_{t}(t)\;$ and AIF was passed to the network to learn the underlying pharmacokinetic parameters, forming a training dataset consisting of a million simulated pairs.

**Figure 5:**
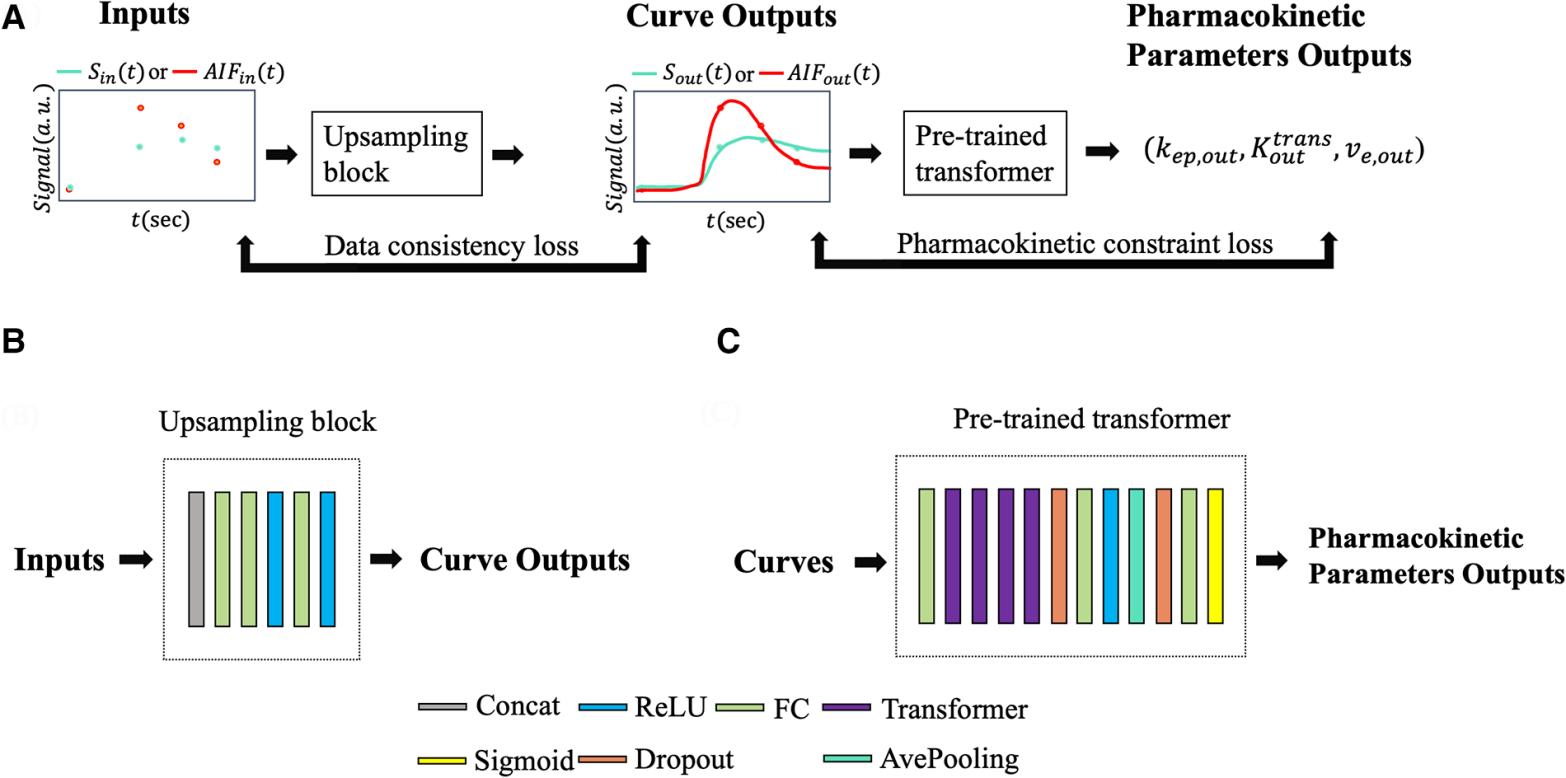
(**A**) Input and output data flow. $S_{\mathrm{in}}$ or $\mathrm{AI}F_{\mathrm{in}}$ was passed to the upsampling block resulting in a temporally super-resolved signal curve $S_{\mathrm{out}}$ or $AIF_{\mathrm{out}}$. The upsampling outputs, along with $\mathrm{AI}F_{\mathrm{out}}$, were passed through the pre-trained pharmacokinetics quantification network to output pharmacokinetic parameters $K_{\mathrm{out}}^{\mathrm{trans}}$, $v_{e,\mathrm{out}}$, and $k_{\mathrm{ep},\mathrm{out}}$. Data consistency loss and pharmacokinetics constraint loss were applied during training. (**B**) Illustration of the upsampling block. (**C**) Illustration of the pre-trained pharmacokinetics quantification network architecture.

The architecture of the pharmacokinetics quantification network is presented in Figure 5C. The network utilized a mixed loss function, combining mean absolute error (MAE) and mean absolute percentage error (MAPE), expressed as:

L=λ⋅MAE+(1−λ)⋅MAPE

Here, the weight λ was empirically chosen as 0.998 to balance the two loss terms. We trained the network with the Adam optimizer (21), setting an initial learning rate of 10^−3^. After 10 initial epochs, we reduced the learning rate by a factor of e^−0.1^ per epoch. The batch size was set to 64, and the network was trained for a total of 100 epochs. Implementation was carried out using Tensorflow (22) on a Linux workstation using a single Nvidia GeForce RTX 2080 TI GPU.

### 2.3. The pharmacokinetics-informed super-resolution network

A pharmacokinetics-informed deep learning-based pipeline was constructed for temporal super-resolution and parameter quantification. The reference signal was the synthesized high-temporal resolution T1W signal, as described before, and the input was downsampled from the reference at timings corresponding to the clinical protocol. Thus, the neural network converts a temporally-downsampled input signal to a temporally super-resolved output signal. After the output signal was determined, pharmacokinetic parameters were estimated using the standard Tofts model.

Figure 5 illustrates the neural network architecture and the pipeline. The upsampling block converted synthesized signals with a 30-s temporal resolution, $S_{\mathrm{in}},$ to a temporally-super-resolved signal $S_{\mathrm{out}}$. Subsequently, $S_{\mathrm{out}}$ and the reference AIF $\left(\mathrm{AI}F_{\mathrm{ref}}\right)$ were input into the pre-trained quantification network described in Section 2.2 to output $k_{\mathrm{ep},\mathrm{out}}$ and $K_{\mathrm{out}}^{\mathrm{trans}};\;\;v_{e,\mathrm{out}}$ was then calculated as $K_{\mathrm{out}}^{\mathrm{trans}}/k_{\mathrm{ep},\mathrm{out}}$.

A combination of mean squared error, data consistency loss, and pharmacokinetic constraint loss was used as the loss function. The data consistency loss was used as a regularization term to enforce the data fidelity between the input samples and the corresponding samples of the outputs at the clinically available phases. The pharmacokinetic constraint loss was additionally used as regularization to leverage known pharmacokinetic models during super-resolution training.

In brief, the cost function was:

$$L=||S_{\mathrm{out}}-S_{\mathrm{ref}}|{|}_{2}^{2}+\lambda_{1}||S_{\mathrm{in}}-\Omega S_{\mathrm{out}}|{|}_{2}^{2}+\lambda_{2}||S_{\mathrm{out}}-\;f_{\mathrm{Tofts}}(\mathrm{AI}F_{\mathrm{out}},k_{\mathrm{ep},\mathrm{out}},K_{\mathrm{out}}^{\mathrm{trans}})|{|}_{2}^{2}$$

With standard Tofts model $f_{\mathrm{Tofts}}(C_{p}(t),k_{\mathrm{ep}},K^{\mathrm{trans}})=$ $K^{\mathrm{trans}}C_{p}(t)*e^{-k_{\mathrm{ep}}t}$ and temporal down-sampling operator Ω. The weighting factors $\lambda_{1}$ and $\lambda_{2}\;$ were empirically determined to be 0.1, which achieved a good balance between different loss terms.

The network was trained with the Adam optimizer (21) with an initial learning rate of 10^−3^. The learning rate was reduced by a factor of e^−0.1^ per epoch after 10 initial epochs. The training was finished after 100 epochs. Voxel-wise training was used with a batch size of 1. The models have been implemented in Tensorflow (22) and trained on a Linux workstation using a single Nvidia GeForce RTX 2080 TI GPU.

A variation of the neural network was trained independently with same settings to validate the effect of pharmacokinetic constraint loss, namely RoQ without pharmacokinetic constraint. The cost function was:

$$L=||S_{\mathrm{out}}-S_{\mathrm{ref}}|{|}_{2}^{2}+\lambda_{1}||S_{\mathrm{in}}-\Omega S_{\mathrm{out}}|{|}_{2}^{2}$$

### 2.4. Performance assessment and statistical analysis

#### 2.4.1. Assessment of the pharmacokinetics quantification network

The performance of the pharmacokinetics quantification network was evaluated using both simulated data and *in-vivo* data. For both scenarios, two classic fitting methods were deployed for comparison: classic non-linear least-square fitting, and least-square fitting with variable projection (VARPRO) (23). Within the simulated data, the agreement between the estimated and true parameters was measured by the coefficient of determination (*R*^2^), the intraclass correlation coefficient (ICC), and the coefficient of variation (CV), with these metrics averaged across all three parameters. Additionally, the normalized root-mean-squared-error (NRMSE) between the predicted curve and the ground truth curve, as well as the average processing time for every 1,000 samples, was examined. For *in-vivo* data, where ground truth parameters were unavailable, only NRMSE and the averaged elapsed time were assessed.

#### 2.4.2. Cross-validation

Ten-fold cross-validation was employed to evaluate the alignment between RoQ pharmacokinetic parameters and those derived with 2 s temporal resolution Multitasking DCE (used as a reference). In each cross-validation cohort, approximately 40 of the 45 subjects were utilized as training data, and the remainder as validation data. Each fold was structured to contain roughly the same number of pixel data. The cross-validation was conducted in such a way that each data fold served once as validation data, with all statistical analyses performed exclusively on this subset.

#### 2.4.3. Assessment of agreement between retrospective and reference pharmacokinetic maps

The agreement between RoQ pharmacokinetic parameters and the reference values was thoroughly assessed. Four temporal-interpolation methods were contrasted: (1) constant interpolation, (2) linear interpolation, (3) RoQ without pharmacokinetics constraint during training, and (4) RoQ itself. The NRMSE between the temporal-interpolated curves and the reference was evaluated, and additional metrics including *R*^2^, ICC, and CV were utilized to gauge the agreement between the estimated and reference parameters. For the proposed RoQ method, a Bland-Altman analysis was additionally carried out for individual ROIs.

#### 2.4.4. Assessment of ability in differentiating abnormal tissues

The capacity of the deep learning-generated pharmacokinetic maps to distinguish abnormal tissues was analyzed through an unpaired *t*-test at a significance level of *P* = .05 across various ROI groups. The pharmacokinetic parameters were averaged within specific ROIs. For healthy volunteers and CP patients, the ROI was the entire pancreas (marked as control and CP, respectively), while in PDAC patients, the ROIs consisted of the tumor and non-tumor parenchymal regions within the pancreas (labeled as tumor and non-tumor). These ROI masks were carefully delineated by an experienced radiologist.

#### 2.4.5. Assessment of the sensitivity to bolus arrival time displacement

Although the bolus arrival time estimated by the test bolus is generally considered accurate (24), minor variations can occur due to the small volume of the test bolus. To account for this, we constructed scenarios by shifting the sampling timings of the input curves within a span of −10 s–+8 s. This simulates the arterial phase being either slightly slower or faster than the actual phase, while keeping the other phases aligned to conserve the temporal gap relative to the simulated arterial phase (as depicted in Figure 1C). Both the NRMSE of the temporal super-resolved curves and the agreement (*R*^2^, ICC, CV) between the RoQ estimated parameters, considering time displacement and reference parameters without it, were assessed.

## 3. Results

**Figure 6:**
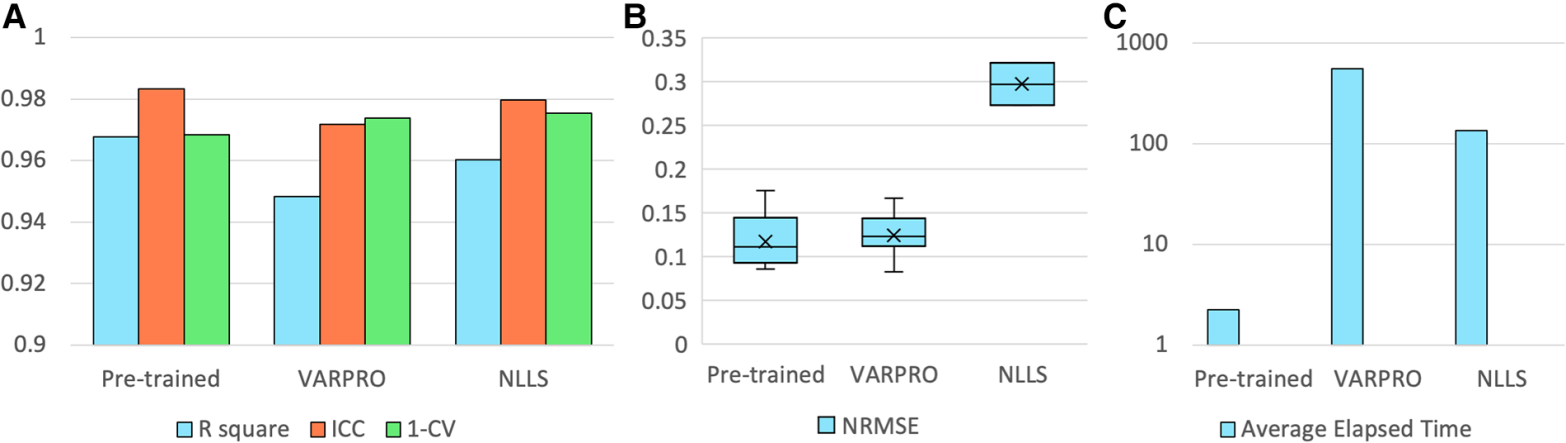
Results of the pre-trained pharmacokinetics quantification network in simulated data. (**A**) The *R*^2^, ICC, and 1-CV of pre-trained network, VARPRO, and NLLS (left to right, respectively) compared to the ground truth. Each metric was averaged over the three pharmacokinetic parameters. (**B**) NRMSE of pre-trained network, VARPRO, and NLLS (left to right, respectively) between the fitted curve by the predicted parameters and the ground truth curve. (**C**) Average elapsed time for estimating 1,000 samples of pre-trained network, VARPRO, and NLLS (left to right, respectively).

**Figure 7:**
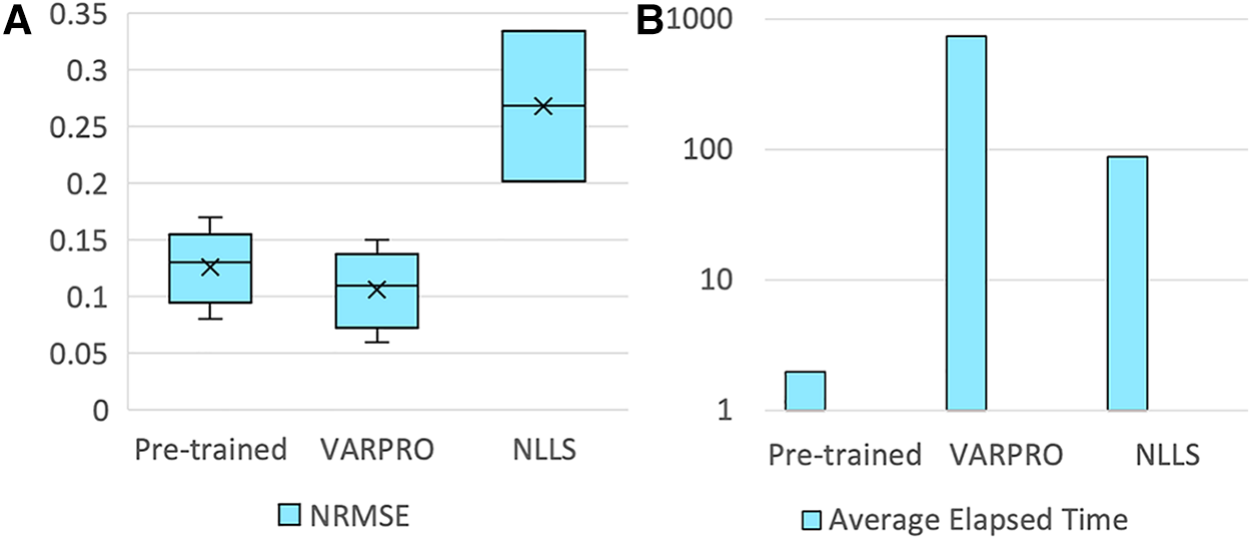
Results of the pre-trained pharmacokinetics quantification network in *in-vivo* data. (**A**) NRMSE of pre-trained network, VARPRO, and NLLS (left to right, respectively) between the fitted curve by the predicted parameters and the reference curve. (**C**) Average elapsed time for estimating one subject of pre-trained network, VARPRO, and NLLS (left to right, respectively).

The pre-trained pharmacokinetics quantification network was initially assessed. Figure 6A reveals that, for simulated data, the network outperformed both VARPRO and NLLS in terms of *R*^2^ and ICC and achieved a CV comparable to those methods. Figure 6B illustrates a respectable NRMSE for the network, comparable to VARPRO and superior to NLLS. Most notably, the network's inference speed was substantially faster than VARPRO and NLLS, as demonstrated in Figure 6C. For *in-vivo* data, the network showed an NRMSE similar to VARPRO but much better than NLLS, as depicted in Figure 7A, along with a significantly quicker estimation, seen in Figure 7B.

**Figure 8:**
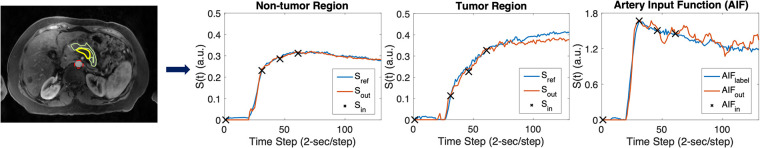
Representative temporal super-resolution example from a 57-year-old PDAC patient. In the upper left image, the tumor mass is marked by a yellow boundary on the synthesized arterial phase image and the abdominal aorta is depicted by a red dashed circle. The non-tumor pancreas body is outlined by a green boundary. In the right three plots, the synthesized multi-phasic DCE-MRI signals used as inputs are shown as black crosses, whereas the network output and reference (2-s temporal-resolution Multitasking DCE data) signals are shown as blue curves and red curves, respectively. The curves plot temporal signal S(t) vs. time step (2-s/step).

Our ten-fold cross-validation experiments further underscored the proposed method's efficiency. Representative examples of high-temporal-resolution DCE curves in a PDAC patient estimated from synthesized multi-phasic DCE-MRI using deep learning are shown in Figure 8. The estimated DCE signal curves closely resemble those of the high-temporal-resolution reference for all three ROIs, aside from some deviations which occur in the extrapolation region after the three synthesized post-contrast phases.

**Figure 9:**
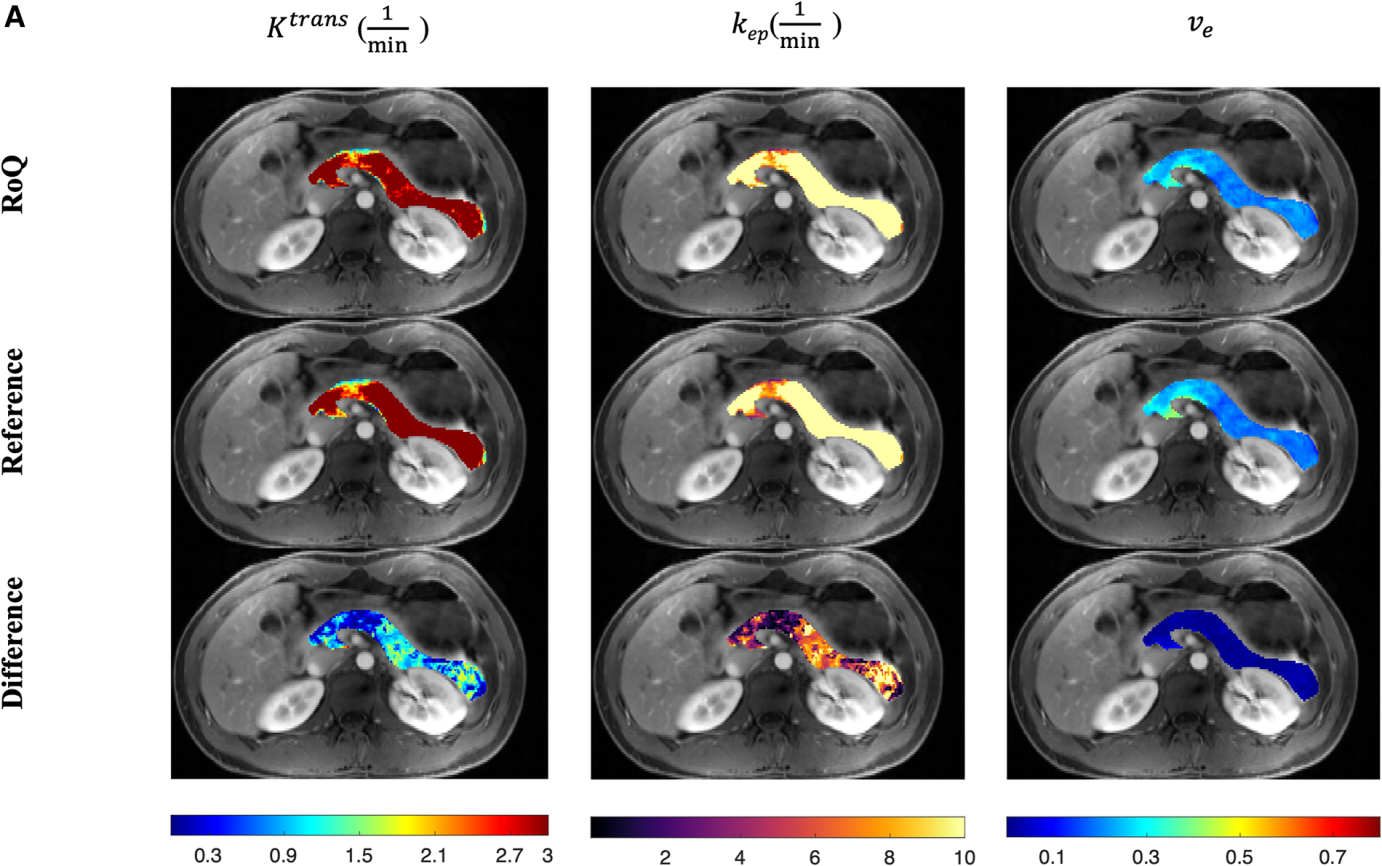
Pancreatic pharmacokinetic mapping in one healthy control. The gray-scale images display the anatomical structure of a representative slice at the arterial phase. The overlaid colored voxels show the pharmacokinetic maps from RoQ (top) and Multitasking DCE reference maps (bottom). Top row: retrospectively estimated $K^{\mathrm{trans}}$ (left), $k_{\mathrm{ep}}$ (middle) and $v_{e}$ (right). Bottom row: reference $K^{\mathrm{trans}}$ (left), $k^{\mathrm{ep}}$ (middle) and $v_{e}$ (right).

**Figure 10:**
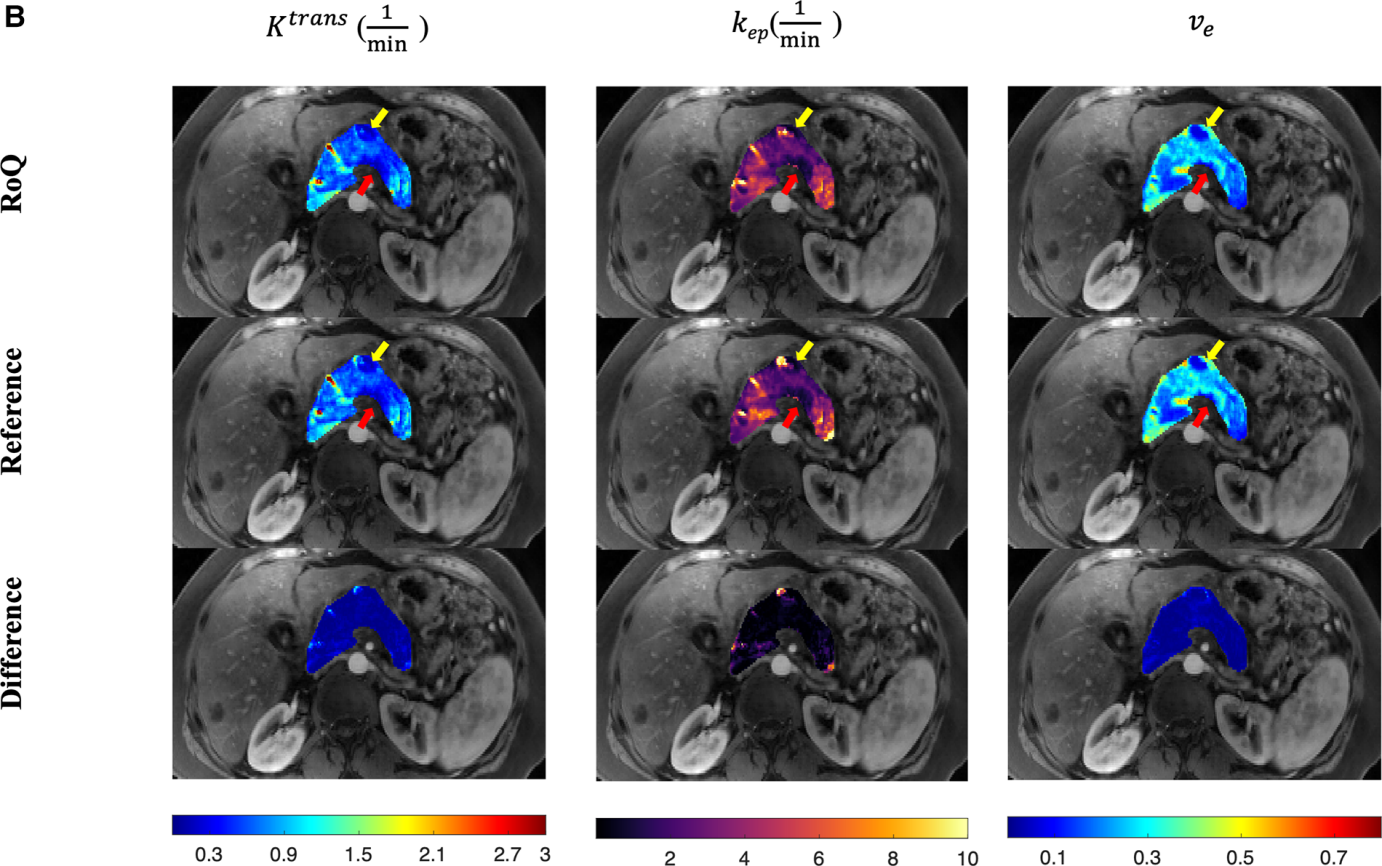
Pancreatic pharmacokinetic mapping in PDAC subject. The gray-scale images display the anatomical structure of a representative slice at the arterial phase. The overlaid colored voxels show the pharmacokinetic maps from RoQ (top) and Multitasking DCE reference maps (bottom). The yellow arrow points out a benign cyst while the red arrow points out the tumor mass. Top row: retrospectively estimated $K^{\mathrm{trans}}$ (left), $k_{\mathrm{ep}}$ (middle) and $v_{e}$ (right) by the neural network. Bottom row: reference $K^{\mathrm{trans}}$ (left), $k^{\mathrm{ep}}$ (middle) and $v_{e}$ (right).

**Figure 11:**
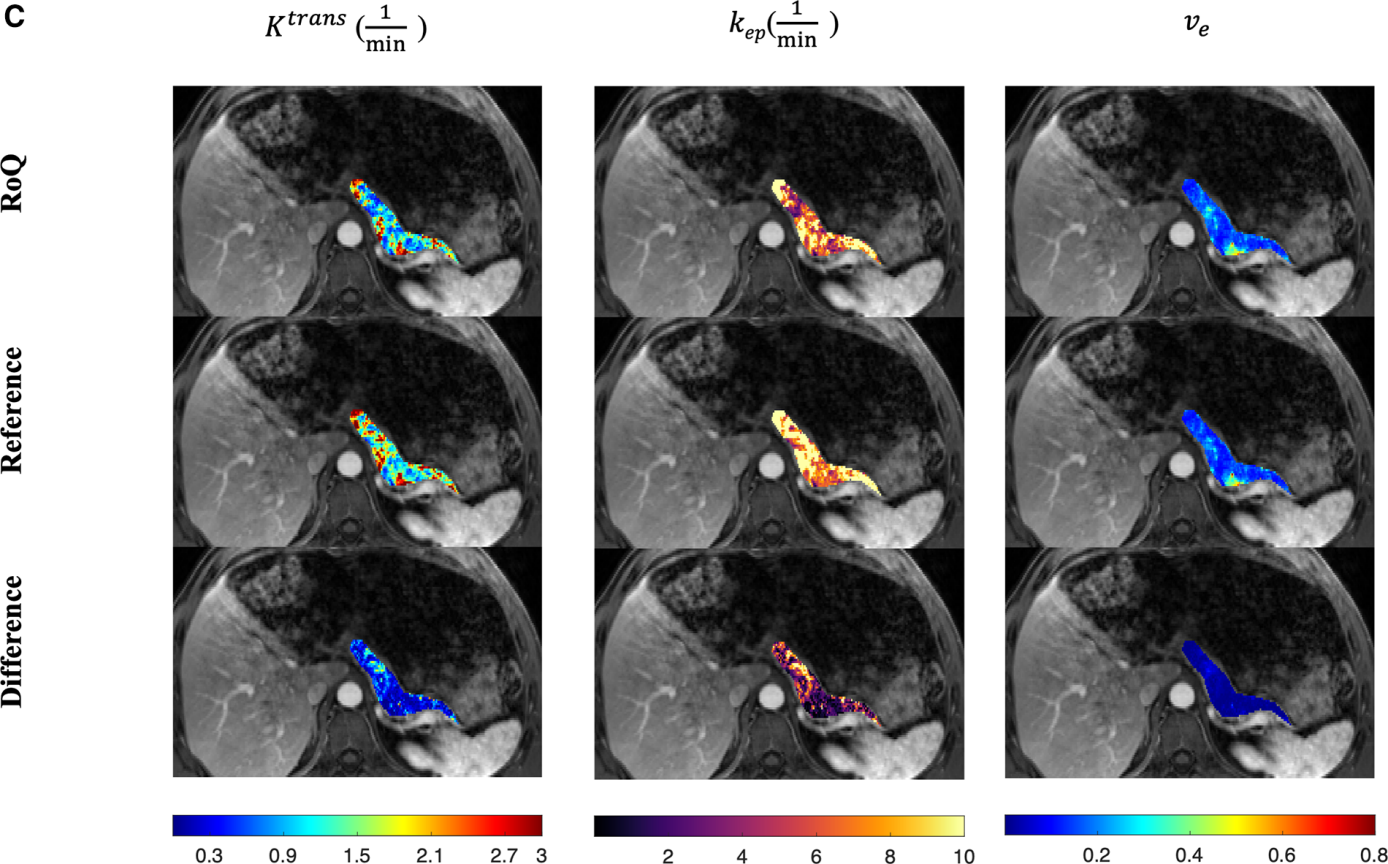
Pancreatic pharmacokinetic mapping in one CP patient. The gray-scale images display the anatomical structure of a representative slice at the arterial phase. The overlaid colored voxels show the pharmacokinetic maps from RoQ (top) and Multitasking DCE reference maps (bottom). Top row: retrospectively estimated $K^{\mathrm{trans}}$ (left), $k_{\mathrm{ep}}$ (middle) and $v_{e}$ (right) by the proposed method from low temporal resolution data. Bottom row: reference $K^{\mathrm{trans}}$ (left), $k^{\mathrm{ep}}$ (middle) and $v_{e}$ (right).

One example of RoQ pharmacokinetic maps and reference pharmacokinetic maps from a healthy control is shown in Figure 9. One example from the same PDAC patient is shown in Figure 10. The delineation of the tumor and cyst by the proposed method resembles the reference very well. One example from a CP patient is shown in Figure 11. In all the examples, pharmacokinetic maps estimated by the proposed RoQ pharmacokinetic maps excellently resemble the reference maps.

**Figure 12:**
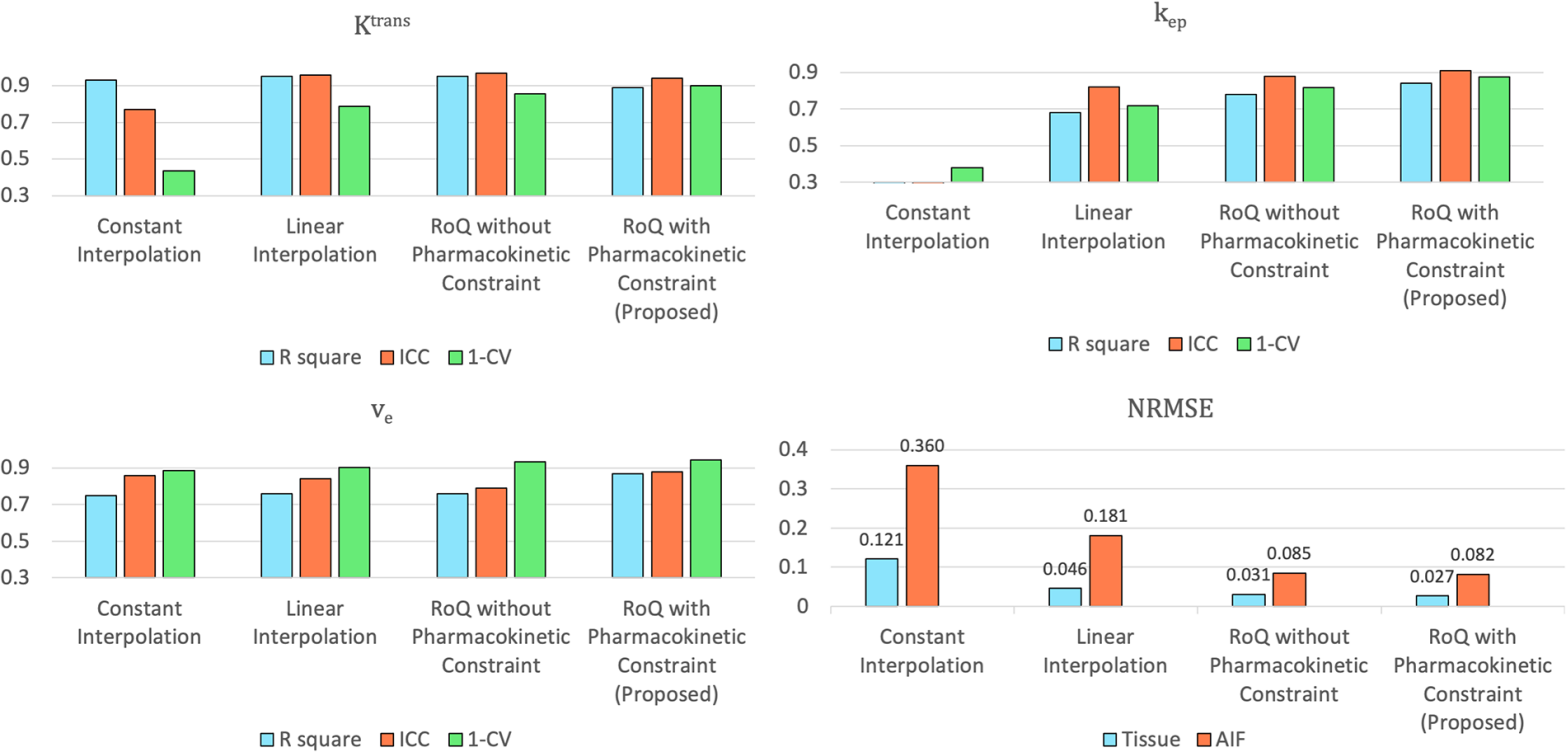
Assessment of pharmacokinetic parameters estimated from different temporal interpolation methods (upper left: $K^{\mathrm{trans}}$, upper right: $k_{\mathrm{ep}}$, lower left: $v_{e}$) and NRMSE of the temporal interpolated curves (lower right).

**Figure 13:**
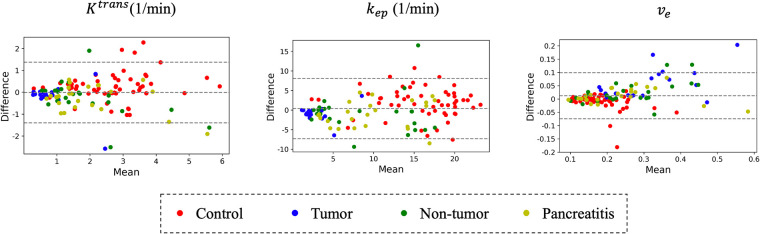
Bland-Altman plot: comparison of RoQ pharmacokinetic parameters vs. reference values for different regions and patient types.

Figure 12 compares different temporal interpolation methods, including constant interpolation, linear interpolation, RoQ without pharmacokinetic constraint, and RoQ. Three of them, linear interpolation, RoQ without pharmacokinetic constraint, and RoQ showed good estimation for $K^{\mathrm{trans}}$ with similar metrics in *R*^2^ and ICC. Moreover, RoQ outperformed the other two methods for higher *R*^2^, ICC, and 1-CV in $k_{\mathrm{ep}}$, $v_{e}$, as well as smaller NRMSE. These results indicated a smaller difference in temporal interpolation by RoQ, thus better parameter agreement against the reference. Specifically, the *R*^2^ and ICC between RoQ pharmacokinetic parameters and the reference ranged between 0.84–0.94, with corresponding CVs of 10.1% for $K^{\mathrm{trans}}$, 12.3% for $k_{\mathrm{ep}}$, and 5.6% for $v_{e}$. A Bland-Altman plot, which visualizes the agreement of these methods using ROIs distinguished by different colors, is illustrated in Figure 13.

**Figure 14:**
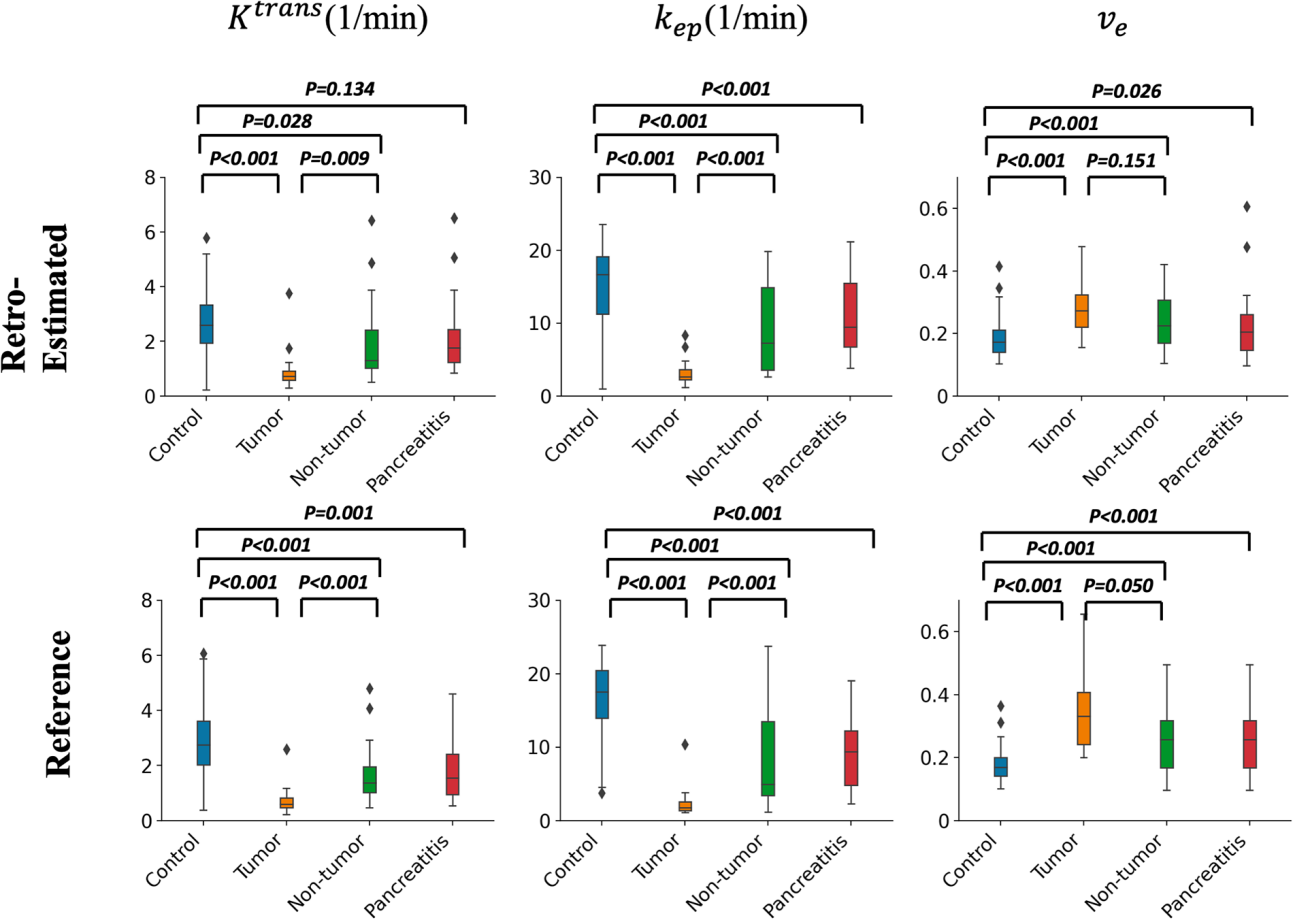
Box plots of pharmacokinetic parameters within different ROIs. Top figure: group comparison of the RoQ pharmacokinetic maps. Bottom figure: group comparison of reference parameters. Left to right: $K^{\mathrm{trans}}$, $k_{\mathrm{ep}}$, $v_{e}$. *P* values from unpaired *t*-tests comparing control vs. tumor, control vs. non-tumor, tumor vs. non-tumor, and control vs. CP were labeled in each box plot.

Figure 14 contains box plots showing the median and quartiles of the RoQ pharmacokinetic parameters (top) and reference parameters (bottom) from the control, PDAC tumor and non-tumor, and CP. Unpaired *t*-tests indicated that all 3 parameters were significantly different (*P* < 0.05) between control and tumor and between control and non-tumor for both the RoQ pharmacokinetic parameters and the reference values. Unpaired *t*-tests further indicated that $K^{\mathrm{trans}}$ and $k_{\mathrm{ep}}$ were significantly different between PDAC tumor and non-tumor, and $k_{\mathrm{ep}}$ and $v_{e}$ were significantly different between the healthy control and CP, for both the RoQ pharmacokinetic parameters and references. $K^{\mathrm{trans}}$ between control and CP and $v_{e}$ between PDAC tumor and non-tumor showed significant differences for the reference values (*P* = 0.001, 0.050, respectively), but did not for the RoQ pharmacokinetic parameters (*P* = 0.134, 0.151, respectively). The directions of differences in all findings were consistent with the references and were in general agreement with published findings (25–28.) To be more specific, compared to the control group, $K^{\mathrm{trans}}$ and $k_{\mathrm{ep}}$ were lower in the PDAC non-tumor, and even lower in the PDAC tumor, whereas $v_{e}$ showed the opposite; compared to the control group, $K^{\mathrm{trans}}$ and $k_{\mathrm{ep}}$ were lower in the CP, while $v_{e}$ showed the opposite.

**Figure 15:**
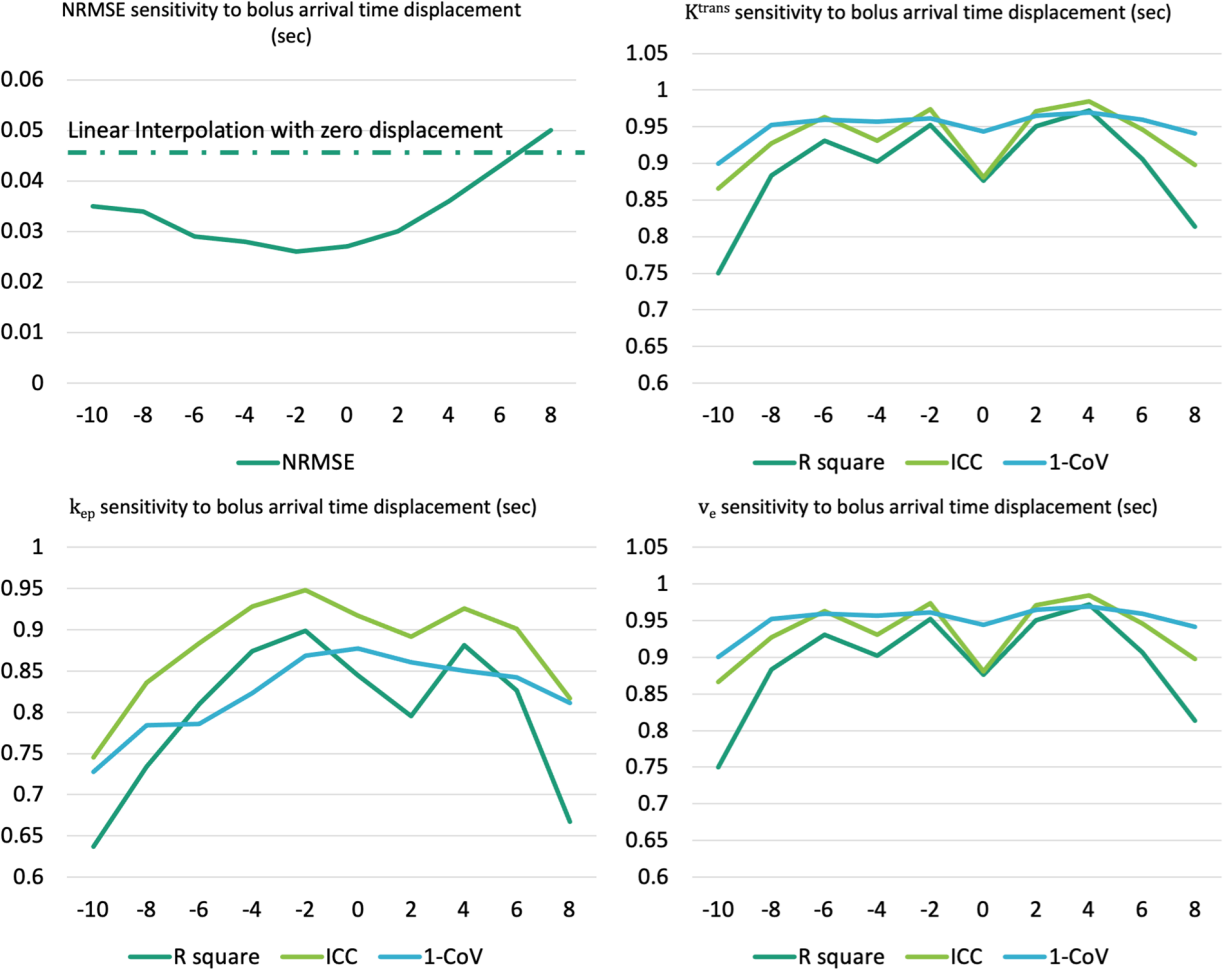
Assessment of sensitivity to the bolus arrival time displacement for NRMSE (upper left) and pharmacokinetic parameters agreement metrics (upper right: $K^{\mathrm{trans}}$, lower left: $k_{\mathrm{ep}}$, lower right: $v_{e}$).

Figure 15 illustrates the NRMSE and parameter agreement metrics’ sensitivity to bolus arrival time displacement. The NRMSE appears relatively robust to this displacement, although an increase in NRMSE is noted with more extended displacement times. Moreover, all of $K^{\mathrm{trans}}$, $k_{\mathrm{ep}}$, $v_{e}$ demonstrate resilience within a narrow range (−6 to +4 s), maintaining reasonable *R*^2^, ICC and CV (lower bound 0.81,0.83, 0.81, respectively).

## 4. Discussion

Quantification of pharmacokinetic parameters in DCE-MRI has important advantages over qualitative imaging as it allows direct characterization of tissue vascularity. A major challenge that limits the broad application of quantitative DCE-MRI in clinical practice is the conflicting requirement of high temporal resolution, high spatial resolution, and sufficient spatial coverage. In addition, body DCE-MRI requires breath-holding to deal with respiratory motion, which further limits the temporal and spatial resolution. Therefore, clinical DCE-MRI typically acquires only three or four post-contrast phases, each with a breath-hold and an around 30-s pause between breath-holds, sacrificing temporal resolution for sufficient spatial resolution and coverage. Due to the limited temporal resolution and the number of phases, DCE signals are usually evaluated qualitatively or semi-quantitatively. Quantitative DCE-MRI techniques have been developed in recent years, such as multiparametric MRI (mpMRI) (6) and Multitasking DCE (7). However, their clinical application is limited because of the need for specialized software, advanced imaging systems, and a lack of rigorous clinical validation at this time.

In this proof-of-concept study, the feasibility of retrospective pharmacokinetic parametric quantification of conventional multi-phasic DCE-MRI using deep learning is demonstrated. Clinical 3-phase DCE-MRI data were synthesized by downsampling high-temporal-resolution multitasking DCE data. Pharmacokinetics-informed based deep learning was used to recover the high-temporal-resolution DCE signal curves for both arterial blood and tissue, which were then used to estimate the pharmacokinetic parameters $K^{\mathrm{trans}}$, $k_{\mathrm{ep}}$, and $v_{e}$ with the standard Tofts model (29). A high level of agreement was found between RoQ pharmacokinetic parameters and reference parameters measured using high-temporal-resolution Multitasking DCE images. RoQ pharmacokinetic parameters also demonstrated the ability to differentiate tumor and non-tumor tissues in pancreatic cancer patients as well as pancreas in CP patients from normal pancreatic tissue in healthy subjects. Upon further validation in a prospective study, this method has the potential to derive pharmacokinetic parameters from clinical multi-phasic DCE images, thus providing the benefits of quantitative DCE-MRI without changing the clinical protocol. This approach may find applications in improving tumor detection, staging, and therapy response prediction.

The proposed scheme had shown better performance than two classic temporal interpolation schemes. Moreover, the pharmacokinetics constraint was proven to benefit both temporal interpolation and parameter estimation. As shown in Figure 12, even though the $K^{\mathrm{trans}}$ metrics were similar, which could be due to the assumption that the arterial phase is well captured, more accurate estimation of $k_{\mathrm{ep}}$, $v_{e}$ was observed in RoQ by adding pharmacokinetics constraint.

RoQ pharmacokinetic parameters had good agreement with those of the 2-s temporal resolution Multitasking DCE reference using *R*^2^, ICC, and CV as evaluation metrics. However, CVs were relatively large for $K^{\mathrm{trans}}$, $k_{\mathrm{ep}}$ (10.1% and 12.3%, respectively). One possible reason could be that the proposed scheme was voxel-wise, and it, therefore, did not take into consideration of spatial information in the local region. Voxel-wise processing could be more susceptible than image-wise processing to factors such as the partial volume effect, motion, and noise. Datasets with large sample sizes and image-based deep learning may reduce CVs in the future. In particular, spatiotemporal networks which also consider spatial information in model prediction (30), and the attention mechanism-based networks which already show great performance in various image processing tasks (31), are promising directions for further improving the current work.

In the ROI-based comparison shown in Figure 7, $K^{\mathrm{trans}}$ between control and CP and $v_{e}$ between PDAC tumor and non-tumor showed significant differences for the reference but did not for the retrospectively estimated. One potential reason for these inconsistencies could be the insufficient data, given the overall percentage of tumor voxels in the whole dataset were small, and the CP patients cohort is small. A prospective study with a larger sample size may help further clarify.

In this study, the standard Tofts model was chosen as the pharmacokinetic model in the application of abdominal imaging, as supported by the literature (19). The main reason was its simplicity and robustness. Furthermore, pancreatic cancer tumors, typically characterized by mild to medium vascularization and hypo-intensity, align well with this model. Models with more compartments and parameters are alternative options, but performance may suffer from the limited number of phases of clinical multi-phasic DCE-MRI. Different models may be more suitable for other applications such as breast (32) and vessel walls (33) which have different vascularity properties (34) and imaging protocols.

The pre-trained pharmacokinetics quantification network was employed for the direct estimation of pharmacokinetic parameters, and its performance in both simulated and *in-vivo* data revealed prompt and precise parameter estimation. Furthermore, the pharmacokinetics constraint within this network was proven to enhance the temporal super-resolution process, underlining its potential value.

A bolus arrival time displacement was simulated to test the robustness of the proposed method. Within a modest range of displacement time, the method demonstrated resilience, maintaining a reasonable NRMSE for temporally super-resolved curves and consistent agreement metrics for parameters. This finding underscores the potential applicability and reliability of the method, even in scenarios where slight variations in bolus arrival times may occur.

The $K^{\mathrm{trans}}$ and $k_{\mathrm{ep}}$ values calculated in this work were on the order of those reported in literature (7, 19), whereas the $v_{e}$ values calculated in this work were in general lower. One potential reason could be the pharmacokinetic model selection, in which case using a different model (e.g., extended Tofts or two-compartment exchange model) may perform more similarly. This also could be due to the relatively short imaging duration (260-s), especially because the difference was seen in $v_{e}$, which is associated with late enhancement. However, given the short imaging duration of the clinical abdominal DCE-MRI protocol, it is challenging to extrapolate over even longer durations.

The clinical imaging protocol used in this study was based on that used routinely at our institution, so the trained deep learning model in this study is currently limited to that imaging protocol. A different imaging protocol will require the training of a new deep learning model. Future work will explore the option of integrating imaging parameters (such as TR/TE, temporal resolution, and the number of phases) as additional inputs to the network for more general application to multiple protocols.

There were several limitations in this study. First, we only used retrospectively synthesized multi-phasic DCE-MRI in training and validation. However, conducting a prospective study in which reference Multitasking and clinical DCE-MRI are sequentially acquired is challenging in practice because of the slow gadolinium washout and the limited contrast dose allowed in each imaging session. A potential solution could be to use a low-dose Multitasking DCE protocol as the reference, which has been shown to allow accurate DCE quantification in breast cancer imaging (32). This would conceivably allow deep learning to train on prospective data, overcoming this limitation. Secondly, the dates of MR imaging and clinical diagnosis have a gap of up to one or two years, so the diagnosis report could be inaccurate at the time of imaging. Therefore, the tumor and non-tumor labels may not be accurate and might lead to errors in quantitative analysis. Most importantly, the dataset was relatively small. A dataset with a larger cohort would be desired in a prospective study to validate this approach in the future.

## 5. Conclusion

A deep learning-based approach was developed for the retrospective quantification of pharmacokinetic parameters by improving the temporal resolution of clinical abdominal DCE-MRI. The retrospective quantitative parameters were capable of differentiating normal pancreas and abnormal pancreas, including PDAC and CP. Tumor delineation was well-preserved in estimated parameter maps. Upon further validation in a prospective study, this technique has the potential to unlock the benefits of quantification of conventional DCE-MRI retrospectively in cancer imaging.

## Data Availability

The raw data supporting the conclusions of this article will be made available by the authors, without undue reservation.
